# Daily Administration of Agmatine Reduced Anxiety-like Behaviors and Neural Responses in the Brains of Male Mice with Persistent Inflammation in the Craniofacial Region

**DOI:** 10.3390/nu17111848

**Published:** 2025-05-28

**Authors:** Yuya Iwamoto, Kajita Piriyaprasath, Andi Sitti Hajrah Yusuf, Mana Hasegawa, Yoshito Kakihara, Tsutomu Sato, Noritaka Fujii, Kensuke Yamamura, Keiichiro Okamoto

**Affiliations:** 1 Division of Oral Physiology, Faculty of Dentistry, Niigata University Graduate School of Medical and Dental Sciences, Niigata 951-8514, Japan; 2 Division of Dental Clinical Education, Faculty of Dentistry, Niigata University Graduate School of Medical and Dental Sciences, Niigata 951-8514, Japan; 3 Department of Restorative Dentistry, Faculty of Dentistry, Naresuan University, Phitsanulok 650000, Thailand; 4 Department of Oral and Maxillofacial Surgery, Faculty of Dentistry, Hasanuddin University, Makassar 90245, Indonesia; 5 Division of General Dentistry and Dental Clinical Education Unit, Niigata University Medical and Dental Hospital, Niigata 951-8514, Japan; 6 Division of Dental Pharmacology, Faculty of Dentistry, Niigata University Graduate School of Medical and Dental Sciences, Niigata 951-8514, Japan; 7 Sakeology Center, Niigata University, Niigata 950-2181, Japan; 8 Department of Agriculture, Faculty of Agriculture, Graduate School of Science and Technology, Niigata University, Niigata 950-2181, Japan

**Keywords:** anxiety, craniofacial tissues, epigenetic change, inflammation

## Abstract

**Background/Objectives**: Chronic craniofacial inflammation is recognized as a factor in anxiety-like behaviors, yet effective therapeutic options remain limited. Agmatine, a dietary bioactive compound found in fermented foods such as sake lees, exhibits modulatory effects on neural functions, alleviating psychological distress like anxiety associated with local inflammation. **Methods**: We investigated both the therapeutic and preventive effects of agmatine on anxiety-like behaviors and the related neural basis in a mouse model of persistent craniofacial inflammation induced by complete Freund’s adjuvant (CFA). **Results**: Comprehensive behavioral assessments, including the elevated plus maze, open field, dark–light box, social interaction, and novel object recognition tests, revealed that therapeutic agmatine administration (1.0 and 30 mg/kg) significantly reduced CFA-induced anxiety-like behaviors, with the higher dose showing more robust and sustained effects across multiple time points. These behavioral improvements were paralleled by reductions in acetylated histone H3, FosB, and c-Fos expression in key anxiety-related brain regions, suggesting a reversal of craniofacial inflammation-associated neural changes. In contrast, preventive agmatine treatment exerted modest and time-dependent behavioral benefits with minimal molecular normalization. Notably, preventive agmatine did not affect general locomotor activity (indicated by total movement distance), indicating that its anxiolytic effects were not confounded by altered locomotor activity. Metabolomic analysis confirmed the presence of agmatine in sake lees (~0.37 mM), supporting the hypothesis that fermented food products might offer dietary routes to emotional resilience. **Conclusions**: These findings underscore agmatine’s promise as a context-specific epigenetic modulator capable of mitigating anxiety-like behaviors by normalizing inflammation-driven molecular dysregulation in the brain.

## 1. Introduction

Agmatine is naturally synthesized by the decarboxylation of arginine [1]. While agmatine is endogenously produced in the mammalian brain, it is also found in a variety of dietary sources. Given its dietary origin and capacity to penetrate the blood–brain barrier [2,3], agmatine has attracted growing attention as a neuroactive compound with potential modulatory effects on emotion [4] and might play a role in mitigating various neurological disorders, particularly anxiety and depression [5,6].

Preclinical investigations have demonstrated that agmatine modulates multiple neurotransmitter systems, including glutamatergic pathways, leading to anxiolytic and antidepressant effects [7,8]. Agmatine is found abundantly in certain fermented beverages such as sake [9,10]. In our previous work, we reported that sake and its byproduct, sake lees (sakekasu), reduced depression-like behaviors in rats [11]. Although those effects might be partially due to agmatine, its presence in the sake lees used at that time was not analytically confirmed [11].

In the current study, our primary objective was to evaluate the nutraceutical properties of agmatine itself on psychological distress, including anxiety-like conditions associated with local inflammation. Notably, chronic inflammation is frequently accompanied by anxiety and depression [12,13], as often seen in conditions like rheumatoid arthritis and temporomandibular disorders (TMDs) [14,15]. These disorders often involve a bidirectional interaction between nociception associated with inflammation and psychological distress [16,17].

In the scope of TMDs, chronic craniofacial pain is associated with anxiety and depression. While pharmacological approaches remain standard, growing interest has emerged in nutraceutical interventions [18,19]. Nutritional neuroscience has highlighted food-derived substances, like polyphenols and omega-3 fatty acids, for their dual roles in regulating inflammation and psychological conditions [20]. Within this framework, agmatine stands out due to its potential to modulate brain functions associated with emotional regulation, especially anxiety and depression [5,8]. However, no studies have directly investigated agmatine’s effect on anxiety-like behaviors and the related brain responses under conditions of persistent craniofacial inflammation. This gap is especially relevant, as chronic TMD involves both local inflammation and impaired emotional processing in the brain [21].

This preclinical study aimed to determine whether daily administration of agmatine can alleviate anxiety-like behaviors and normalize associated neural changes in a mouse model of persistent craniofacial inflammation. To create localized inflammation that resembles a TMD, we used a proven model that involves injecting complete Freund’s adjuvant (CFA) into the masseter muscle [22,23,24].

To assess anxiety-like behaviors, mice were evaluated at multiple time points using a battery of behavioral assays, including the elevated plus maze (EPM), dark–light box (DL), open field (OF), and social interaction (SI) tests, which have been utilized in our previous studies [25,26,27]. In addition, cognitive function was tested with the novel object recognition (NOR) test [26,28]. These evaluations were chosen based on emerging clinical and preclinical findings suggesting that peripheral inflammatory conditions can increase anxiety-like behaviors and impair cognition [29]. Various rodent models of orofacial pain have demonstrated impairment in learning and working memory [30].

To elaborate on neural mechanisms mediating the anxiolytic-like effects of agmatine, we evaluated molecular markers of epigenetic changes and neural activation in the brain. These included histone H3 acetylation and immediate early gene expression (c-Fos, FosB) in key brain regions because changes in the level of these markers have been associated with not only anxiety-like responses but also nociception in the periphery [26,31,32]. These brain regions included the anterior cingulate cortex (ACC), insular cortex (IC), rostral ventromedial medulla (RVM), and upper cervical spinal dorsal horn (C2), a network known to be altered in chronic inflammatory states accompanied by anxiety-like conditions [33,34,35].

This study bridges nutritional neuroscience and craniofacial inflammation-associated anxiety-like responses by investigating agmatine as a functional dietary compound with both therapeutic and prophylactic potential. To link our findings back to dietary sources, we also investigated the presence of agmatine in sake lees, a traditional Japanese fermented food, to determine its potential role as a functional component [11]. While the functional effects of sake lees were not directly assessed here, this compositional insight provides a supplementary angle on agmatine from food-based sources.

## 2. Materials and Methods

### 2.1. Animals

In total, 118 male C57BL/6J adult mice and 4 male Institute of Cancer Research (ICR) mice (Charles River Laboratories) were utilized. All protocols of animal experimentation were evaluated and sanctioned by the Intramural Animal Care and Veterinary Science Committee of Niigata University (Permit Number: SA01380), and were conducted following the established Guiding Principles for the Care and Use of Laboratory Animals (National Institutes of Health). Upon arrival, the C57BL/6J mice were 6 weeks old and were permitted to acclimatize to their cages for a minimum duration of 1–2 weeks, facilitating their adaptation to the new environment. The 6-week-old ICR mice were maintained in solitary environmental conditions. The mice were accommodated in transparent acrylic cages (30 × 20 × 15 cm) and were provided with unrestricted access to regular pelletized diet and water. The housing environment was regulated for temperature and humidity, and it adhered to a 12 h light/dark cycle (light phase: 7:00–19:00). The precise number of animals employed for every experimental analysis is documented in Supplementary Tables S1 and S2.

### 2.2. Persistent Craniofacial Inflammatory Model (CFA Mice)

Mice were subjected to anesthesia through the administration of 0.3 mg/kg of medetomidine (Domitor; Nippon Zenyaku Kogyo, Koriyama, Fukushima, Japan), 4.0 mg/kg of midazolam (Midazolam; Sandoz, Kamiyama, Yamagata, Japan), and 5.0 mg/kg of butorphanol (Vetorphale; Meiji Seika Pharma, Tokyo, Japan) via intraperitoneal injection. Complete Freund’s adjuvant (CFA, Mycobacterium tuberculosis, Sigma-Aldrich, St Louis, MO, USA) suspended in an oil/saline solution (0.9% sodium chloride, WAKO, Osaka, Japan) (1:1) was employed as the inflammatory agent. CFA (5 µL) was administered into the left masseter muscle region utilizing a 26-gauge needle [36]. Indeed, our preliminary experiments revealed increases in craniofacial pain-like behaviors in mice twelve days after the masseter muscle injection of CFA (Supplemental Figure S1).

### 2.3. Experimental Design

Anxiety-like behaviors were assessed using the elevated plus maze (EPM), dark–light (DL), and social interaction (SI) tests on Days 1, 3, 7, and 10 following CFA injection. Additionally, open field (OF) and novel object recognition (NOR) tests were conducted on Days 2, 4, 8, and 11. These data were compared with those measured before CFA injection (the Pre level). Upon completion of the behavioral experiments, animals were subjected to immunohistochemistry (IHC) analysis on Day 12. Mice receiving no CFA injection were employed as controls (non-CFA group).

### 2.4. Agmatine (AGM) Treatment

**Figure 1:**
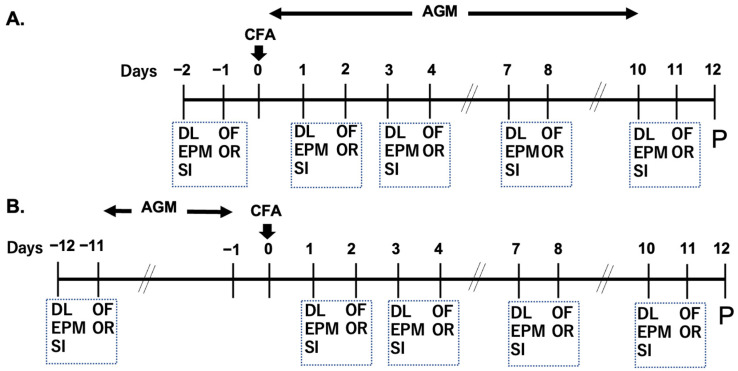
Experimental protocols. On Day 0, complete Freund’s adjuvant (CFA) was injected into the left masseter muscle to induce persistent craniofacial inflammation. Agmatine (AGM) was injected intraperitoneally, either initiating after CFA injection (therapeutic protocol, AGMt, (**A**)) or before CFA injection (preventive protocol, AGMp, (**B**)). Behavioral assessments, including DL, OF, EPM, SI, and OR, were conducted from Day −2 to Day 11 in Protocol A and from Day −12 to Day 11 in Protocol B. On Day 12, all mice underwent perfusion. Abbreviations: AGM, agmatine; CFA, complete Freund’s adjuvant; DL, dark–light test; OF, open field test; EPM, elevated plus maze test; SI, social interaction test; OR, novel object recognition (NOR) test; P, perfusion.

The therapeutic (Figure 1A) and preventive (Figure 1B) effects of agmatine sulfate (agmatine, TCL Development Co., Ltd., Tokyo, Japan; 1.0 mg/kg or 30 mg/kg, intraperitoneal (IP) administration) on anxiety-like responses were evaluated using behavioral and immunohistochemical (IHC) procedures. Agmatine was administered via IP injection either from Day 0, immediately after the induction of craniofacial inflammation, to Day 10, or as a preventive treatment from Day −11 to Day −1. The selected agmatine dose has been previously shown to inhibit anxiety-like behaviors in rodents [37,38]. A saline-treated group (1.0 mL/kg) served as the control.

### 2.5. Behavioral Tests

Behavioral test procedures followed the protocols described in our previous studies [25,26]. Prior to the commencement of testing, the mice underwent an acclimatization period of one hour within the testing environment. To ensure unbiased interpretations, the experimenter remained blinded to the test groups. Each different test was conducted separately at least one hour between tests. The results were assessed across different time points (e.g., the Pre level vs. post-CFA injection) and treatment groups (vehicle, 1.0 mg/kg agmatine, and 30 mg/kg agmatine in each time course).

#### 2.5.1. Elevated Plus Maze (EPM) Test

A mouse was centrally positioned within the maze, which comprised two open and two closed arms. This apparatus was elevated approximately 50 cm from the floor (PM-DR25M-S, Brain Science Idea Co., Ltd., Osaka, Japan). The cumulative time spent by the subject in the open arms was recorded over a five-minute period.

#### 2.5.2. Dark–Light (DL) Test

A mouse was positioned within a dark–light box consisting of two compartments, a light and a dark chamber, interconnected via an aperture. Initially, the subjects were introduced into the light chamber, where their behavior was monitored over 15 min. The time spent within each compartment was quantified through digital counters equipped with infrared sensors (SCANET MV-40 MOV, MELQUEST Co., Ltd., Toyama, Japan).

#### 2.5.3. Social Interaction (SI) Test

Mice were introduced into the center of a transparent acrylic cage, which served as an open field area (45 × 45 × 30 cm) with a black frosted Plexiglas floor. The expansive area was delineated into two distinct regions: interaction zone (IZ) and corner zone (CZ). The SI test consisted of two consecutive sessions, each lasting 2.5 min. In the initial session, mice explored the open field in the absence of a male stranger mouse (an Institute of Cancer Research (ICR) mouse, 30–35 g) within the IZ. Subsequently, following a one-minute interval, the ICR mouse was introduced into the center of the IZ, enclosed within a perforated acrylic apparatus. Across both sessions, the cumulative time spent in the IZ was noted utilizing digital counters with infrared sensors (SCANET MV-40 MOV, MELQUEST Co., Ltd., Toyama, Japan). The SI test was performed no less than two hours after the completion of the DL test.

#### 2.5.4. Open Field (OF) Test

Mice were housed in a transparent acrylic cage featuring a black frosted Plexiglas base (45 × 45 × 30 cm) for 5 min. The cumulative distances of movement and the duration spent within the center region were quantified using digital counters equipped with infrared sensors (SCANET MV-40 MOV, MELQUEST Co., Ltd., Toyama, Japan).

#### 2.5.5. Novel Object Recognition (NOR) Test

NOR serves as a quantifiable indicator of recognition memory and primarily relies on the innate propensity of rodents to allocate a greater duration to exploring an unfamiliar object than a previously encountered one [26,39,40]. The NOR test comprises two trials. During the initial trial, subjects were introduced into an OF arena and permitted to engage with two akin objects (familiar objects), positioned in the A and A’ zones, for 5 min prior to returning to the home cage. Following a period of 60 min, the subjects were reintroduced to the experimental setting and presented with a familiar object (in the “A area”) and a new object (in the “B area”) during the second trial. On the day designated for testing, the duration spent in each area was recorded in both trials (5 min each) utilizing digital counters with infrared sensors (SCANET MV-40 MOV, MELQUEST Co., Ltd., Toyama, Japan). The NOR test was conducted at least one hour after the OF test. Data collected from each treatment over the time course were compared to the Pre level. In this test, we assessed the effects of 30 mg/kg agmatine on behavioral responses.

### 2.6. Immunohistochemical (IHC) Experiments

The IHC experiments determined the effects of agmatine on the neural activities indicated by c-Fos and FosB expressions, as well as those on the epigenetic changes indicated by histone H3 acetylation in key areas of the brain associated with anxiety.

#### 2.6.1. Preparations

On the twelfth day, mice were conditioned to profound anesthesia utilizing a combination of three pharmacological agents, specifically formulated with 0.3 mg/kg of medetomidine (Domitor; Nippon Zenyaku Kogyo, Koriyama, Fukushima, Japan), 4.0 mg/kg of midazolam (Midazolam; Sandoz, Kamiyama, Yamagata, Japan), and 5.0 mg/kg of butorphanol (Vetorphale; Meiji Seika Pharma, Tokyo, Japan) administered 24 h following the last behavioral assessments (Figure 1). The perfusion of the mice was conducted via cardiac injection with saline (20 mL), succeeded by the infusion of cold 4% paraformaldehyde in 0.1 M phosphate-buffered saline (PBS, pH = 7.4, 20 mL). The brain, along with the upper cervical spinal cord (C2), was excised and subsequently post-fixed in 4% paraformaldehyde overnight; thereafter, it was preserved in a 30% sucrose solution in 0.1 M PBS at a temperature of 4 °C for several days. Both anatomical regions were sectioned into transverse slices (0.04 mm in thickness) utilizing a freezing microtome (REM-710 Retratome; Yamato, Aasaka City, Saitama, Japan). The collected sections were meticulously transferred sequentially into multi-well tissue culture plates containing cold 0.1 M PBS, which were for IHC.

#### 2.6.2. IHC Procedures

The methodology pertaining to IHC was delineated comprehensively in our prior publications [27]. In summary, the tissue sections underwent multiple washes with PBS, followed by incubation with affinity-purified rabbit anti-mouse c-Fos (9F6, 1:1500 Cell Signaling Technology, Danvers, MA, USA) or FosB (1:2000; Abcam, Cambridge, UK) or acetylation of histone H3 (1:2000 Merck, Darmstadt, Germany) antibodies for a duration of 16 h in PBS supplemented with 0.3% Tween 20 and 5% normal goat serum at 4 °C, subsequently treated with biotinylated goat anti-rabbit IgG antibody (1:200; Vector Laboratories, Newark, CA, USA) for 2 h at ambient temperature, and finally exposed to avidin–biotin–peroxidase complex (Vector Laboratories, Newark, CA, USA) for 1 h. Following the visualization of the c-Fos, FosB, and acetylation of histone H3 immunoreactivities through incubation in diaminobenzidine and nickel solution catalyzed by 0.01% peroxidase, all sections were thoroughly washed with Tris-buffered saline, affixed to glass slides, and allowed to dry. Subsequently, sections adhered to the glass slides were subjected to dehydration using ethanol, cleared with xylene, and cover-slipped.

#### 2.6.3. Data Analysis

The immunoreactivities of c-Fos, FosB, and histone H3 acetylation were discerned according to the observations of uniform black–gray components exhibiting distinctly defined margins under bright-field illumination. Sections were delineated by the prominent anatomical landmark within each region, utilizing the atlas of Franklin and Paxinos, 2008 [41], alongside our previous reports [25].

In the ACC and IC (1.3 mm to 1.6 mm rostral to the Bregma) [25,42,43], the findings were articulated as the mean count of c-Fos-, FosB-, and acetylation of histone H3-positive cells. These measurements were exclusively derived from the left hemisphere, as the quantity of immune-positive cells on the left was comparable to that on the right within the CFA-injected side.

The quantification of positive cells was performed within the delineated regions of the anterior portions of ACC (0.3 × 0.6 mm) and IC (0.3 × 0.5 mm) utilizing the Image J software (version 1.53; National Institutes of Health, USA). In the regions of RVM (1.0 × 0.4 mm) and C2 (0.5 × 0.3 mm), the quantification of c-Fos-, FosB-, and acetylation of histone H3-positive cells was conducted employing Image J (National Institutes of Health). The quantification of positive cells within the RVM and C2 regions was executed at −6.0 mm and −6.2 mm caudal to the Bregma, and 5.0 mm caudal to the Obex, respectively [41]. Images were obtained at a magnification of ×10 and averaged across the right and left hemispheres in each subject. Acetylated histone H3-, FosB-, and c-Fos-positive nuclei were classified as indicative signals demonstrating grayscale contrast levels exceeding 125 units (the total possible range was 0–255) [44]. Cells were then counted automatically, with those exceeding 40 pixels being defined as cells within the region of interest [45].

Two sections were chosen at random, and the mean values per section in each area were computed for every individual animal. Subsequently, the collective mean and standard deviation (SD) were measured. Notably, specific immunoreactivities for each primary antibody were found to be completely absent following the exclusion of the primary antibody. The evaluators who undertook the quantification of the immunoreactivities were unable to distinguish the experimental groups.

### 2.7. Statistical Analysis

Statistical investigations were performed utilizing SPSS Statistics (version 21.0; IBM, Armonk, NY, USA) for behavioral and IHC experiments. Data concerning behavioral responses and the quantification of acetylated histone H3, c-Fos, and FosB were subjected to analysis of variance (ANOVA) or two-way ANOVA as necessary. The findings are articulated as the mean ± SD. Post hoc analyses comprising each comparison were executed employing the Bonferroni test. A statistical significance threshold of *p* < 0.05 was established. The examiners who conducted the statistical analysis were blinded to the respective treatment groups.

### 2.8. Metabolomic Analysis of Sake Lees Extract

Sake lees was solubilized in PBS at a final concentration of 0.5 g/mL, subsequently filtered through a 0.22 µM filter, and thereafter underwent ultrafiltration utilizing Ultrafree-MC from HMT (Human Metabolome Technologies Inc., Yamagata, Japan). The resultant sake lees extract was subjected to analytical examination employing capillary electrophoresis time-of-flight mass spectrometry (CE-TOFMS), utilizing the Agilent CE capillary electrophoresis system, which is integrated with an Agilent 6230 time-of-flight mass spectrometer (Agilent Technologies, Inc., Santa Clara, CA, USA), in accordance with previously delineated methodologies [46,47]. The systems were operated through the Agilent MassHunter Workstation Data Acquisition (Agilent Technologies) and interconnected via a fused silica capillary (50 μm i.d. × 80 cm total length) utilizing commercial electrophoresis buffers (H3301-1001 and I3302-1023 for cation and anion analyses, respectively, HMT) as the electrolyte. The spectrometer was calibrated to scan within the mass-to-charge ratio (*m*/*z*) range of 50 to 1000, with peak data extracted employing MasterHands, an automatic integration software (Keio University, Tsuruoka, Yamagata, Japan) to obtain peak-specific information encompassing the *m*/*z*, peak area, and migration time [24]. Signal peaks that were indicative of isotopomers, adduct ions, and other derivative ions of recognized metabolites were systematically excluded, and the remaining peaks were annotated in accordance with HMT’s metabolite database predicated upon their respective *m*/*z* values and MTs. The areas corresponding to the annotated peaks were subsequently normalized against internal standards and the sample mass to derive the relative quantifications of each metabolite. The quantification of agmatine was executed based on one-point calibrations utilizing a standard reference compound.

## 3. Results

### 3.1. Anxiety-like Behaviors

#### 3.1.1. Elevated Plus Maze (EPM) Test

**Figure 2:**
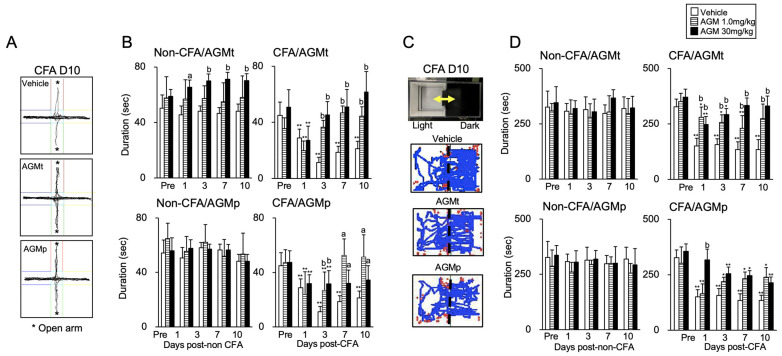
Impact of therapeutic (AGMt) and preventive (AGMp) agmatine (AGM) administration on anxiety-like behavior in the elevated plus maze (EPM, (**A**,**B**)) and dark–light (DL, (**C**,**D**)) tests. (**A**) Representative movement trajectories of mice during the EPM test on Day 10 comparing CFA mice treated with the vehicle, AGMt (30 mg/kg), or AGMp (30 mg/kg). (**B**) Summary of AGMt and AGMp effects on time spent in the elevated open arms. (**C**) Representative movement trajectories of mice during the DL test on Day 10 comparing CFA mice treated with the vehicle, AGMt (30 mg/kg), or AGMp (30 mg/kg). (**D**) Summary of AGMt and AGMp effects on time spent in the light room. * *p* < 0.05, ** *p* < 0.0001 versus pre-treatment within each group; a, *p* < 0.05, b, *p* < 0.0001 versus the vehicle group on the corresponding day.

Figure 2A displays example trajectories of a mouse with CFA-induced craniofacial inflammation assessed by the EPM test on Day 10. AGMt- and AGMp-treated mice exhibited greater movement in the elevated open arms compared to the vehicle-treated mice. Figure 2B shows the summary data for the effects of AGMt and AGMp on the time spent in the elevated open arms.

- Non-CFA mice (Figure 2B, left panel)

There was no significant main effect of the time course (days post-non-CFA) in either the AGMt (F(4, 100) = 0.32, *p* = 0.87) or AGMp (F(4, 104) = 0.9, *p* = 0.46) groups. On the other hand, treatment displayed a significant main effect in the AGMt (F(2, 25) = 10.9, *p* < 0.0001) and AGMp (F(2, 26) = 23.7, *p* < 0.0001) groups.

- AGMt Group

The AGMt (30 mg/kg) group spent significantly more time in the elevated open arms compared to the vehicle group on Days 1 (*p* < 0.05), 3, 7, and 10 (*p* < 0.0001 for all except Day 1). In contrast, the time spent in the elevated open arms in the AGMt (1.0 mg/kg) group was comparable to that of the vehicle-treated group (*p* > 0.1).

b. AGMp Group

Post hoc analysis revealed an absence of significant differences in the time allocated to the elevated open arms when comparing the AGMp and vehicle groups across the time course (*p* > 0.1 for all time points).

- CFA mice (Figure 2B, right panel)

- AGMt Group

There were significant main effects of the time course (F(4, 108) = 23.6), *p* < 0.0001) and treatment (F(2, 27) = 101.2, *p* < 0.0001). In the vehicle group, the time spent in the elevated open arms on Days 1, 3, 7, and 10 was significantly lower than the pre-CFA injection levels (Pre, *p* < 0.0001 for all comparisons). In contrast, for both the 1.0 mg/kg and 30 mg/kg AGMt groups, a significant decrease in the time spent within the elevated open arms was observed only on Day 1 (*p* < 0.0001). On Days 3, 7, and 10, the AGMt group (both doses) spent significantly more time in the elevated open arms compared to the vehicle group (*p* < 0.0001 for all comparisons).

b. AGMp Group

There were significant main effects of the time course (F(4, 112) = 24.5), *p* < 0.0001) and treatment (F(2, 28) = 22.1, *p* < 0.0001). In both the 1.0 and 30 mg/kg AGMp groups, the time spent in the elevated open arms on Days 1 and 3 was significantly lower than pre-CFA injection levels (vs. Pre, *p* < 0.0001 for both time points). However, the time spent in the elevated open arms on Days 7 and 10 in both the AGMp groups was not significantly different from the Pre level (*p* > 0.1). Both AGMp groups spent significantly more time in the elevated open arms compared with vehicle-treated mice on Days 3 (*p* < 0.0001), 7 (*p* < 0.05), and 10 (*p* < 0.05).

#### 3.1.2. Dark–Light Room (DL) Test

Figure 2C shows example trajectories of a mouse with CFA-induced craniofacial inflammation assessed in the DL test on Day 10. Both the AGMt- and AGMp-treated mice exhibited greater movement in the light room compared to the vehicle-treated mice. Figure 2D summarizes the effects of AGMt and AGMp on anxiety-like behaviors in the DL test.

1. Non-CFA mice (Figure 2D, left panel)

There was a small but significant main effect of time course in the AGMt (F(4, 100) = 2.81, *p* < 0.03) or AGMp (F(4, 104) = 3.028, *p* = 0.02) groups. However, post hoc analysis showed no significant changes in time spent in the light chamber across time points compared to the Pre level. There was no significant main effect of treatment in the AGMt group (F(2, 25) = 2.5, *p* < 0.1). In contrast, AGMp treatment displayed a significant main effect (F(2, 26) = 6.57, *p* < 0.005), but a post hoc test showed no significant differences between the vehicle, 1.0 mg/kg, and 30 mg/kg AGM treatments (*p* > 0.05 for both).

2. CFA mice (Figure 2D, right panel)

Significant main effects of the time course and treatment were noted in both the AGMt (F(4, 108) = 9.75, *p* < 0.0001, F(2, 27) = 10.06, *p* < 0.0001) and AGMp (F(4, 112) = 5.243, *p* = 0.001, F(2, 28) = 32.56, *p* = 0.0001) groups, respectively.

- AGMt Group

In the vehicle group, the time spent in the light room was significantly reduced on Days 1, 3, 7, and 10 compared to the pre-CFA injection level (*p* < 0.0001 for all time points). In AGMt group, the time spent in the light room was also significantly reduced on Days 1 (*p* < 0.05), 3 (*p* < 0.05), and 7 (*p* < 0.0001) in the 1.0 mg/kg group and on Days 1 (*p* < 0.0001) and 3 (*p* < 0.05) in the 30 mg/kg group compared to the pre-CFA injection level. However, at these time points, both AGMt groups spent noticeably longer time in the light room than the vehicle group (*p* < 0.0001 for all time points).

b. AGMp Group

The time spent in the light room was significantly lower on Days 1 (*p* < 0.0001), 3 (*p* < 0.05), 7 (*p* < 0.05), and 10 (*p* < 0.05) in the 1.0 mg/kg AGMp group, and on Days 3 (*p* < 0.0001), 7 (*p* < 0.05), and 10 (*p* < 0.0001) in the 30 mg/kg group than the Pre level of each corresponding group. In contrast, the time spent in the light room did not significantly differ between the AGMp and the vehicle groups on Days 3, 7, and 10. However, on Day 1, the 30 mg/kg AGMp group exhibited a significantly longer time in the light room than the vehicle group (*p* < 0.0001).

#### 3.1.3. Open Field (OF) Test

**Figure 3:**
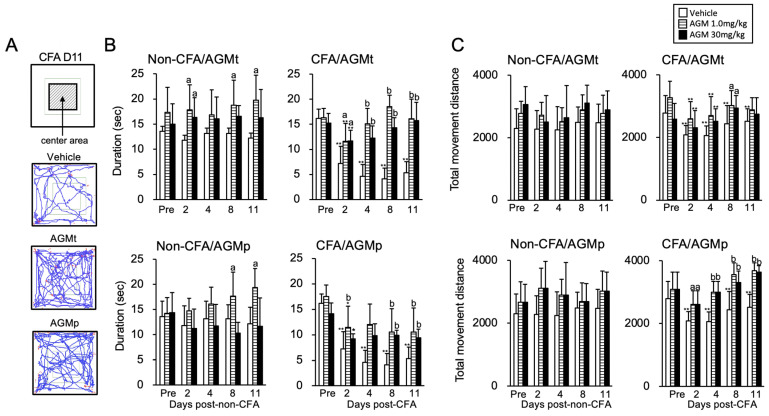
Impact of therapeutic (AGMt) and preventive (AGMp) agmatine (AGM) administration on anxiety-like behavior in the open field (OF) test. (**A**) Representative movement trajectories of mice during the OF test on Day 11, comparing CFA mice treated with the vehicle, AGMt (30 mg/kg), or AGMp (30 mg/kg). The images illustrate movement patterns within the OF arena. In the trajectory plots, red indicates periods when the mouse is standing (rearing or upright posture), blue indicates periods when the mouse is walking or has all four paws on the ground, and green outlines the boundaries of the center area. (**B**) Summary of AGMt and AGMp effects on time spent in the center area. (**C**) Summary of AGMt and AGMp effects on total movement distance. * *p* < 0.05, ** *p* < 0.0001 versus pre-treatment within each group; a, *p* < 0.05, b, *p* < 0.0001 versus the vehicle group on the corresponding day.

Figure 3A shows example trajectories of a mouse with CFA-induced craniofacial inflammation assessed in the test on Day 11. Both the AGMt and AGMp-treated mice exhibited greater movement in the center area compared to the vehicle-treated mice. Figure 3B and Figure 3C summarize the effects of AGMt and AGMp on the time spent within the center area and the total movement distance during a five-minute observation in non-CFA and CFA mice, respectively.

1 1. Time spent in the center area (Figure 3B)

- Non-CFA mice

- AGMt Group

While there was no significant main effect of the time course (F(4, 100) = 0.39, *p* = 0.81), a significant main effect of the treatment was observed (F(2, 25) = 9.12, *p* < 0.0001). Post hoc analysis showed that the AGMt group (1.0 mg/kg) spent significantly more time in the center area than the vehicle group on Days 2, 8, and 11 (*p* < 0.05 for all). Similarly, the AGMt (30 mg/kg) group showed an increased time spent in the center area on Day 2 (*p* < 0.05).

- AGMp Group

There was a significant main effect of the time course (F(4, 108) = 15.5, *p* < 0.0001) and treatment (F(2, 27) = 3.6, *p* < 0.041). Post hoc analysis revealed that the time spent in the center area on Days 8 and 11 in the AGMp (1.0 mg/kg) group was significantly greater than in the Pre period (prior to the start of AGM treatment, *p* < 0.05 for both). However, no significant differences were observed between treatments at any time point (*p* < 0.1).

b. CFA mice

- AGMt Group

There was a significant main effect of the time course (F(4, 108) = 24.78, *p* < 0.0001) and treatment (F(2, 27) = 73.4, *p* < 0.0001). In the vehicle group, the time spent in the center area on Days 2, 4, 8, and 11 was significantly lower than the pre-CFA injection level (Pre) (*p* < 0.0001 for all time points). In both the 1.0 mg/kg and 30 mg/kg AGMt groups, the time spent in the center area was significantly lower on Day 2 than the Pre level (*p* < 0.0001 for both); however, on Days 4, 8, and 11, there was no significant difference from the Pre level (*p* < 0.1). Treatment comparisons revealed that both AGMt groups spent significantly more time in the center area than the vehicle group at all observed time points.

- AGMp Group

There was a significant main effect of the time course (F(4, 112) = 24.5, *p* < 0.0001) and treatment (F(2, 28) = 22.1, *p* < 0.0001). In both the 1.0 mg/kg and 30 mg/kg AGMp groups, the time spent in the center area on Day 2 (*p* < 0.05) was significantly lower than the pre-CFA injection level. However, the time spent in the center area on Days 4, 8, and 11 in both AGMp groups was similar to the Pre level (*p* < 0.1). On Days 8 and 11, the time spent in the center area in both AGMp groups was significantly greater than the vehicle group (*p* < 0.0001) for both. On Day 2, that was significantly greater in the 1.0 mg/kg AGMp group than the vehicle-treated group (*p* < 0.0001).

2 Total movement distance on the OF (Figure 3C)

- Non-CFA mice

- AGMt Group

There was a significant main effect of the time course (F(4, 108) = 2.24, *p* < 0.008) and treatment (F(2, 27) = 15.34, *p* < 0.001). However, post hoc tests revealed no significant differences between the Pre level and any time point (*p* < 0.1), nor between the vehicle and both AGMt groups (*p* < 0.1).

- AGMp Group

There was significant main effect of the time course (F(4, 108) = 3.65, *p* < 0.008) and treatment (F(2, 27) = 15.35, *p* < 0.001). Post hoc tests revealed no significant effect of these.

b. CFA mice

- AGMt Group

There was a significant main effect of the time course (F(4, 116) = 6.18, *p* < 0.0001) and treatment (F(2, 28) = 4.061, *p* < 0.0001). In the vehicle group, the total movement distance on Days 2, 4, 8, and 11 was significantly lower than the pre-CFA injection (Pre) level (*p* < 0.0001 for all time points). Similarly, in both the 1.0 and 30 mg/kg AGMt groups, the total movement distance was significantly reduced on Days 2 and 4 compared to the Pre level (*p* < 0.0001 for both time points). Comparisons between treatment groups revealed significant increases in the total movement distance in both AGMt groups compared with the vehicle-treated group on Day 8 (*p* < 0.05).

- AGMp Group

There was a significant main effect of the time course (F(4, 112) = 3.545, *p* < 0.009) and treatment (F(2, 28) = 40.39, *p* < 0.0001). While in both the 1.0 mg/kg and 30 mg/kg AGMp groups, the total movement distance on Days 2, 4, 8, and 11 was not significantly different from the Pre level, the total movement distance on all time points in both AGMp groups was significantly greater than in the vehicle-treated group (*p* < 0.05 for Day 2, *p* < 0.0001 for Days 4, 8, and 11).

#### 3.1.4. Social Interaction (SI) Test

**Figure 4:**
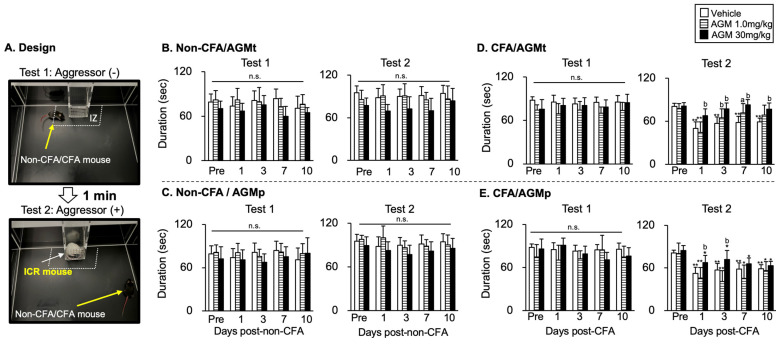
Impact of therapeutic (AGMt) and preventive (AGMp) agmatine (AGM) administration on the social interaction (SI) behaviors using the SI test. (**A**) An illustration of the SI field is presented, along with the interaction zone (IZ) used in the SI test. A non-CFA or CFA mouse was placed within the field in the absence (Test 1, upper panel) and presence (Test 2, lower panel) of an aggressor mouse for 2.5 min each. Test 2 was conducted 1 min after the completion of Test 1. Data summary of the effect of AGMt (**B**,**D**) and AGMp (**C**,**E**) on the time spent within the IZ on each testing day in non-CFA and CFA mice, respectively. * *p* < 0.05, ** *p* < 0.0001 versus pre-treatment within each group; a, *p* < 0.05; b, *p* < 0.0001 versus the vehicle group on the corresponding day; n.s., no significance.

Figure 4A illustrates the SI arena, showing the interaction zone (IZ), where the time spent in the IZ was compared between treatment groups in the absence (Test 1, the upper image) and presence (Test 2, the lower image) of an aggressor. Figure 4B–E summarize the effects of AGMt and AGMp on the time spent within the IZ.

1 Non-CFA mice

- AGMt group (Figure 4B)

There was no significant main effect of the time course (Test 1: F(4, 92) = 1.2, *p* < 0.3, Test 2: F(4, 92) = 1.36, *p* < 0.26). A significant main effect of the treatment was observed in both Test 1 (F(2, 23) = 3.7, *p* < 0.04) and Test 2 (F(2, 23) = 5.2, *p* < 0.007). However, post hoc tests revealed no significant differences between the treatments for each time course.

b. AGMp Group (Figure 4C)

There was no significant main effect of the time course (Test 1: F(4, 104) = 0.78, *p* < 0.5, Test 2: F(4, 104) = 0.2, *p* < 0.1). A main effect of treatment was not observed in Test 1 (F(2, 26) = 2.1, *p* < 0.15), but was observed in Test 2 (F(2, 26) = 10.7, *p* < 0.001). However, subsequent post hoc analyses demonstrated an absence of statistically significant variations among the treatments in Test 2 for each time course.

2 CFA mice

- AGMt group (Figure 4D)

There was a significant main effect of the time course (F(4, 112) = 25.9, *p* < 0.0001) in Test 2, but not in Test 1 (F(4, 116) = 0.75, *p* < 0.56). The time spent in the IZ was significantly lower on Days 1, 3, 7, and 10 in the vehicle group (*p* < 0.0001 for all time points), and on Day 1 in the 1.0 mg/kg AGMt group (*p* < 0.0001). In the 30 mg/kg AGMt group, the time spent in the IZ on Days 1, 3, 7, and 10 was not significantly different from that at the Pre level (*p* > 0.05).

A main effect of the treatment was observed in both Test 1 (F(2, 29) = 7.32, *p* < 0.01) and Test 2 (F(2, 28) = 5.8, *p* < 0.0001); however, in Test 1, post hoc tests revealed no significant difference between the treatments in each time course. In Test 2, treatment comparisons at each time point revealed that the time spent in the IZ in the 30 mg/kg AGMt group on Days 1, 3, 7, and 10 was significantly greater than in the vehicle group at each time point (*p* < 0.0001 for all time points), while that in the 1.0 mg/kg AGMt group was significantly greater on Days 3 (*p* < 0.0001) and 7 (*p* < 0.05) than the vehicle group.

b. AGMp group (Figure 4E)

There was a significant main effect of time course (F(4, 112) = 4.64, *p* < 0.02) in Test 1 and Test 2 (F(4, 112) = 31.7, *p* < 0.0001). Post hoc tests revealed no significant difference between the time courses in Test 1 (*p* < 0.1). In Test 2, the time spent in the IZ was significantly lower on Days 1 (*p* < 0.0001), 3 (*p* < 0.0001), 7 (*p* < 0.05), and 10 (*p* < 0.05) in the 1.0 mg/kg AGMp group, and on Days 1, 3, 7, and 10 (*p* < 0.05 for all time points) in the 30 mg/kg AGMp group, respectively, than the Pre level. There was a significant main effect of the treatment (F(2, 28) = 13.3, *p* < 0.0001) in Test 1 and Test 2 (F(2, 28) = 17.9, *p* < 0.0001). Post hoc analysis revealed that in Test 2, the time spent in the IZ on Days 1 (*p* < 0.0001) and 3 (*p* < 0.0001) in the 30 mg/kg AGMp was significantly greater than that in the vehicle-treated CFA group.

#### 3.1.5. Novel Object Recognition (NOR) Test

**Figure 5:**
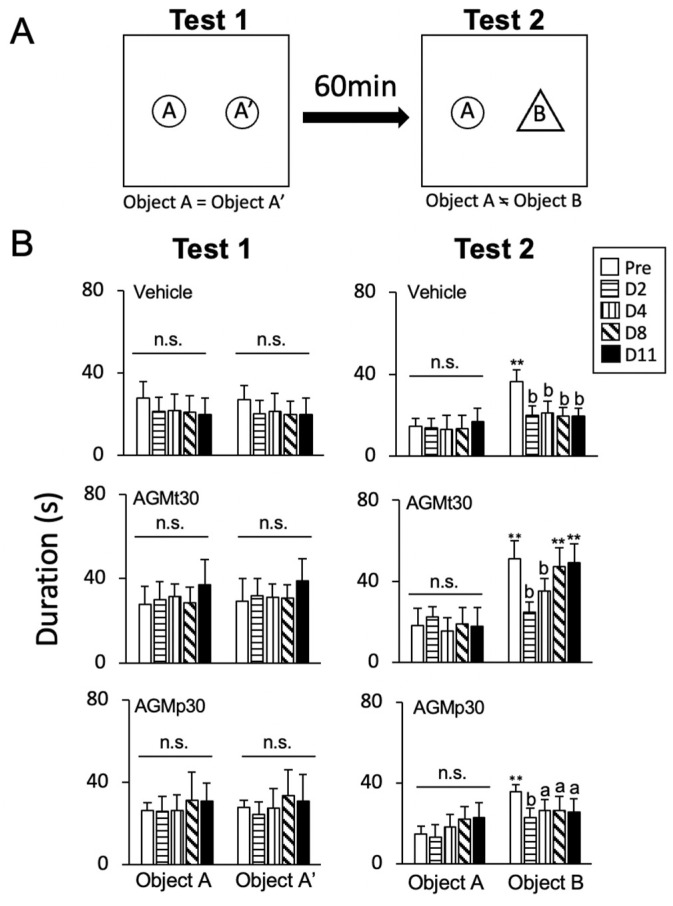
Experimental procedures (**A**) and summary data (**B**) for the effect of agmatine (AGM) on the ability of novel object recognition (NOR) in CFA-evoked persistent craniofacial inflammatory mice. The NOR tests were conducted on Pre and Days 2, 4, 8, and 11 after CFA injection. The average time spent around the objects A and A′ areas in Test 1, and objects A and B in Test 2, in each treatment. ** *p* < 0.0001 versus object A; a, *p* < 0.05; b, *p* < 0.0001 versus Pre on the corresponding day; n.s., no significance.

Figure 5 presents the experimental procedures (A) and summary data (B).

1 Non-CFA mice (Supplement Figure S2)

In each group, while there was no significant main effect of the time course (Pre, Days 2, 4, 8, and 11) in Tests 1 and 2, a significant main effect of region (object) was observed. Post hoc analysis revealed that in Test 2, the time spent around object B was significantly greater than that around object A in each group.

2 CFA mice (Figure 5B)

- Vehicle group

A significant main effect of the time course (F(4, 160) = 9.76, *p* < 0.0001) and region (F(3, 40) = 14.5, *p* < 0.0001) was observed. In Test 2, the time spent around object B on Days 2, 4, 8, and 11 was not significantly greater than that around object A, except at the Pre level (*p* < 0.0001).

b. AGMt group

There was a significant main effect of the time course (F(4, 144) = 11.73, *p* < 0.0001) and region (F(3, 36) = 42.47, *p* < 0.0001). Post hoc analysis revealed that in Test 2, while the time spent around object B on Day 8 (*p* < 0.0001) and Day 11 (*p* < 0.0001) was significantly greater than that around object A (*p* < 0.0001), that on Days 2 and 4 was significantly lower than the Pre level (*p* < 0.0001 for both).

c. AGMp group

There was a significant main effect of the time course (F(4, 144) = 5.3, *p* < 0.0001), and region (F(3, 36) = 14.1, *p* < 0.0001). Post hoc analysis revealed that in Test 2, the time spent around object B was not significantly different from that around object A (*p* > 0.1 for Days 2, 4, 8, and 11), and the time spent around object B was significantly lower than the Pre level on Day 2 (*p* < 0.0001) and Days 4, 8, and 11 (*p* < 0.05 for all time points).

### 3.2. Immunohistochemistry (IHC)

#### 3.2.1. ACC and IC

1 Acetylation histone H3 immunoreactivity

**Figure 6:**
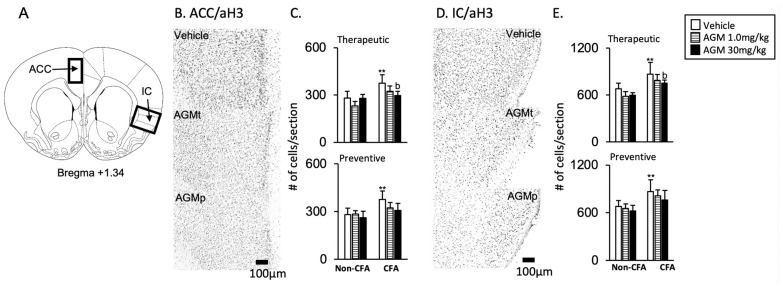
Effects of agmatine on acetylated histone H3 immunoreactivity in the brain cortices in the CFA mice. Representative images illustrating the immunoreactivity of acetylated histone H3 within the anterior portions of the cingulate cortex (ACC) and insular cortex (IC) of CFA-injected mice treated with the vehicle, therapeutic (30 mg/kg, AGMt), or preventive (30 mg/kg, AGMp) administration of agmatine (**A**,**B**,**D**). Cell quantifications were performed inside the enclosed area indicated in panel A. The effects of AGMt and AGMp on the expression of acetylated histone H3 in the ACC are summarized in graphs. (**C**) and IC (**E**). ** *p* < 0.0001 vs. non-CFA vehicle group; b, *p* < 0.0001 vs. CFA vehicle group.

Figure 6A,B,D show schematic diagrams with boxes indicating the areas selected for quantitative analysis. Representative images of acetylated histone H3 immunoreactivity are presented for the vehicle, therapeutic (AGMt), and preventive (AGMp) administration of 30 mg/kg agmatine, focusing on the anterior regions of the ACC and IC. The immunoreactivity is localized in cell nuclei. Of note, in the vehicle-treated group, the number of acetylated histone H3-positive cells in both AGMt and AGMp-treated CFA mice was significantly greater than that in non-CFA mice (*p* < 0.0001, Figure 6).

- AGMt (Figure 6C,E)

There was a significant main effect of the group, with F(5, 39) = 14.6, *p* < 0.0001 in the ACC, and F(5, 39) = 14.7, *p* < 0.0001 in the IC. In the CFA group treated with 30 mg/kg, but not 1.0 mg/kg, of AGMt, the number of acetylated histone H3-positive cells was significantly lower than in vehicle-treated mice in both the ACC and IC (*p* < 0.0001 for both regions).

b. AGMp (Figure 6C,E)

There was a significant main effect of the group, with F(5, 39) = 11.1, *p* < 0.0001 in the ACC, and F(5, 39) = 7.4, *p* < 0.0001 in the IC. However, post hoc tests revealed that the number of acetylated histone H3-positive cells in both AGMp-treated mice was not significantly different from that in vehicle-treated mice.

2 FosB and c-Fos immunoreactivities

**Figure 7:**
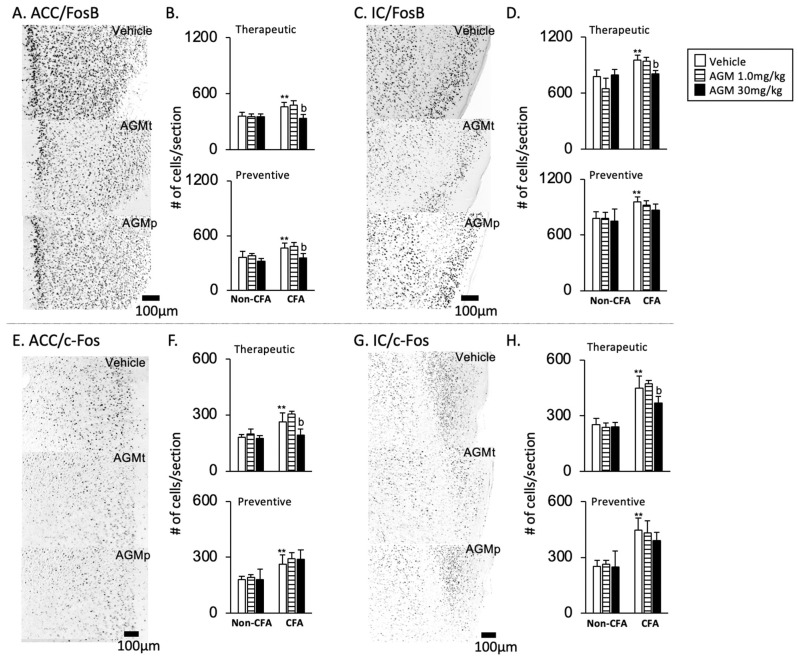
Effects of agmatine on FosB (upper panels) and c-Fos (lower panels) immunoreactivities in the brain cortices. Representative images displaying FosB (**A**,**C**) and c-Fos (**E**,**G**) immunoreactivities in the anterior portions of the cingulate cortex (ACC) and insular cortex (IC) of CFA-injected mice treated with the vehicle, therapeutic (30 mg/kg, AGMt), or preventive (30 mg/kg, AGMp) administration of agmatine. Graphs summarize the effects of AGMt and AGMp on FosB and c-Fos expressions in the ACC (**B**,**F**) and IC (**D**,**H**). ** *p* < 0.0001 vs. non-CFA vehicle group; b, *p* < 0.0001 vs. CFA vehicle group.

Figure 7A,E and Figure 7C,G present representative images of FosB and c-Fos immunoreactivities, respectively, following the vehicle, therapeutic (AGMt), and preventive (AGMp) administration of 30 mg/kg agmatine. The immunoreactivities are localized predominantly within cell nuclei. In the vehicle-treated group, the number of FosB- and c-Fos-positive cells of CFA mice was significantly greater than that in non-CFA mice (*p* < 0.0001).

- FosB (Figure 7A–D)

In the AGMt group, there was a significant main effect of the group for FosB expression, with F(5, 39) = 15.3, *p* < 0.0001 in the ACC, and F(5, 41) = 17.2, *p* < 0.0001 in the IC. In the CFA group treated with 30 mg/kg, but not 1.0 mg/kg, of AGMt, the number of FosB-positive cells was significantly lower than in vehicle-treated mice in both the ACC and IC (*p* < 0.0001 for both regions). Similarly, in the AGMp group, there was a significant main effect of the group in FosB expressions with F(5, 40) = 20.4, *p* < 0.0001 in the ACC, and F(5, 32) = 7.7, *p* < 0.0001 in the IC. The number of FosB-positive cells in the ACC, but not IC, in 30 mg/kg AGMp-treated CFA mice was significantly lower than in vehicle-treated CFA mice.

b. c-Fos (Figure 7E–H)

There was a significant main effect of the group found for c-Fos expression, with F(5, 42) = 9.8, *p* < 0.0001 in the ACC, and F(5, 42) = 40.5, *p* < 0.0001 in the IC. In the CFA group treated with 30 mg/kg, but not 1.0 mg/kg of AGMt, the number of c-Fos-positive cells was also significantly lower than in vehicle-treated mice in both regions (*p* < 0.0001 for both). Similarly, there was a significant main effect of group in c-Fos expressions with F(5, 41) = 14.7, *p* < 0.0001 in the ACC, and F(5, 41) = 28.3, *p* < 0.0001 in the IC. However, post hoc analysis showed that there was no significant difference in the quantity of c-Fos-positive cells in both AGMp-treated CFA mice and the vehicle-treated CFA mice.

#### 3.2.2. RVM and C2

1 Acetylation histone H3 immunoreactivity

**Figure 8:**
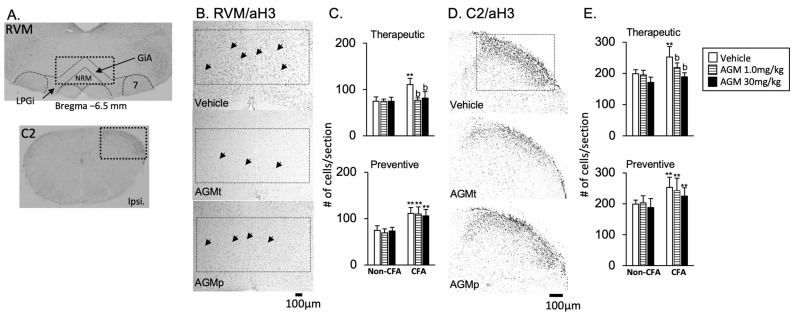
Effects of agmatine on acetylated histone H3 immunoreactivity in the caudal brainstem and spinal dorsal horn. (**A**,**B**,**D**) Representative images show acetylated histone H3 immunoreactivity in the rostral ventromedial medulla (RVM) and upper cervical spinal dorsal horn (C2) of CFA-injected mice following treatment with the vehicle, therapeutic agmatine (30 mg/kg, AGMt), or preventive agmatine (30 mg/kg, AGMp). Cell quantification was performed within the boxed region highlighted in panel A. Graphs illustrate the effects of AGMt and AGMp on acetylated histone H3 expression in the RVM (**C**) and C2 (**E**), respectively. ** *p* < 0.0001 vs. corresponding treatment group in non-CFA group; b, *p* < 0.0001 vs. CFA vehicle group.

Figure 8A,B,D show schematic diagrams with boxes indicating the areas selected for quantitative analysis. Representative images of acetylated histone H3 immunoreactivity are presented for vehicles, therapeutic (AGMt), and preventive (AGMp) administration of 30 mg/kg agmatine, focusing on the RVM and C2 regions. The immunoreactivity is localized in cell nuclei. In the vehicle-treated CFA group, CFA mice had considerably greater numbers of FosB- and c-Fos-positive cells than non-CFA mice. (*p* < 0.0001).

- AGMt (Figure 8C,E, upper panels)

There was a significant main effect of the group with F(5, 41) = 10.7, *p* < 0.0001 in the RVM, and F(5, 41) = 26.4, *p* < 0.0001 in the C2 region. In the CFA group treated with 1.0 mg/kg and 30 mg/kg of AGMt, the number of acetylated histone H3-positive cells was significantly lower than in vehicle-treated mice in both the RVM and C2 regions (*p* < 0.0001 for both regions).

b. AGMp (Figure 8C,E, lower panels)

There was a significant main effect of the group with F(5, 40) = 14.9, *p* < 0.0001 in the RVM, and F(5, 41) = 7.4, *p* < 0.0001 in the C2 region. However, post hoc tests revealed that the number of acetylated histone H3-positive cells in the RVM and C2 regions in both AGMp-treated groups was not significantly different from that in vehicle-treated mice.

2 FosB and c-Fos immunoreactivities

**Figure 9:**
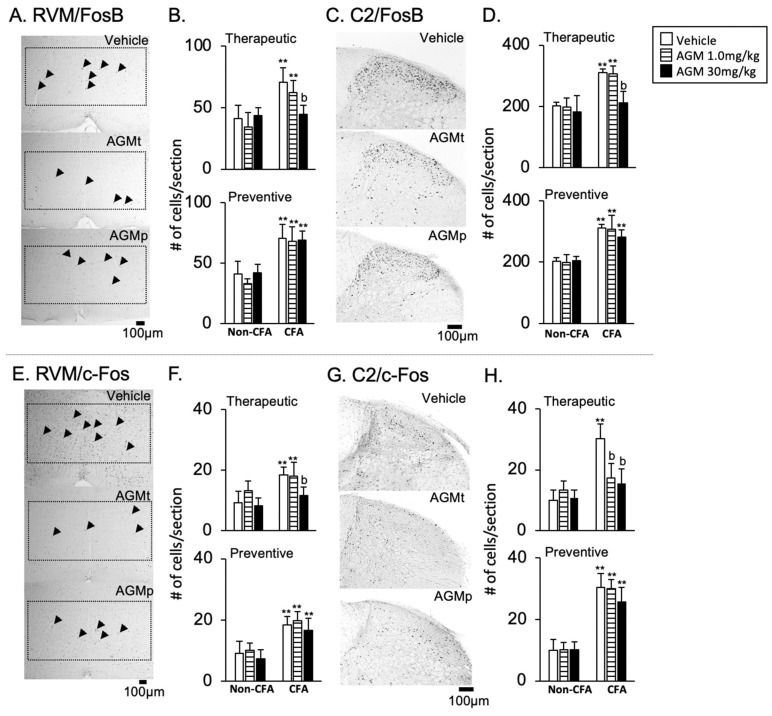
Effects of agmatine on FosB (upper panels) and c-Fos (lower panels) immunoreactivities in the caudal brainstem and spinal dorsal horn. Representative images displaying FosB (**A**,**C**) and c-Fos (**E**,**G**) immunoreactivities in the rostral ventromedial medulla (RVM) and upper cervical spinal dorsal horn (C2) of CFA-injected mice following treatment with the vehicle, therapeutic agmatine (30 mg/kg, AGMt), or preventive agmatine (30 mg/kg, AGMp). Graphs summarize the effects of AGMt and AGMp on FosB (**B**,**D**) and c-Fos (**F**,**H**) expressions in the RVM and C2. ** *p* < 0.0001 vs. corresponding treatment group in non-CFA group; b, *p* < 0.0001 vs. CFA vehicle group.

Figure 9A,E and Figure 9C,G present representative images of FosB and c-Fos immunoreactivity, respectively, following the vehicle, therapeutic (AGMt), and preventive (AGMp) administration of 30 mg/kg agmatine. The immunoreactivities are localized predominantly within cell nuclei. In the vehicle-treated CFA group, the number of FosB- and c-Fos-positive cells of CFA mice was significantly greater than that of non-CFA mice (*p* < 0.0001 for both markers).

- FosB (Figure 9A–D)

In the AGMt group, there was a significant main effect of the group for FosB expression, with F(5, 41) = 23.2, *p* < 0.0001 in the RVM, and F(5, 43) = 14.9, *p* < 0.0001 in the C2 region. In the CFA group treated with 30 mg/kg, but not 1.0 mg/kg, of AGMt, in both the RVM and C2 areas, there were significantly lower numbers of FosB-positive cells than in vehicle-treated mice (*p* < 0.0001 for both regions).

In the AGMp group, there was a significant main effect of the group in FosB expression, with F(5, 40) = 29.3, *p* < 0.0001 in the RVM, and F(5, 40) = 28.9, *p* < 0.0001 in the C2 region. However, post hoc analysis revealed no significant difference in FosB-positive cells between the AGMp- and vehicle-treated CFA groups in both regions.

b. c-Fos (Figure 9E–H)

In the AGMt group, there was a significant main effect of the group for c-Fos expression, with F(5, 41) = 12.3, *p* < 0.0001 in the RVM, and F(5, 41) = 31.5, *p* < 0.0001 in the C2 region.

In the CFA group treated with 30 mg/kg, but not 1.0 mg/kg, of AGMt, the number of c-Fos-positive cells was significantly lower than in vehicle-treated CFA mice in the RVM (*p* < 0.0001). In contrast, in both the 1.0 and 30 mg/kg AGMt groups, the number of c-Fos-positive cells in the C2 region was significantly smaller than in the vehicle-treated CFA group (*p* < 0.0001 for both doses).

In the AGMp group, a significant main effect of the group was also observed, with F(5, 41) = 19.3, *p* < 0.0001 in the RVM and F(5, 41) = 58.8, *p* < 0.0001 in the C2 region. However, the number of c-Fos-positive cells in AGMp-treated mice did not differ significantly from the vehicle-treated CFA group.

### 3.3. Determination of Agmatine in Sake Lees

We conducted a quantitative determination of agmatine in sake lees, a traditional Japanese fermented food. The results revealed that it contained 0.37 mM agmatine.

## 4. Discussion

The primary findings of our research demonstrate that the therapeutic administration of agmatine effectively attenuates anxiety-like behaviors associated with persistent craniofacial inflammation. Consistently, therapeutic administration normalized neural responses, as evidenced by changes in histone H3 acetylation, as well as FosB and c-Fos expression, in key brain regions implicated in anxiety regulation. In contrast, preventive administration led to minor but statistically significant improvements in certain anxiety-like behaviors at specific time points; however, it did not result in significant alterations in these molecular markers. In the subsequent sections, we initially present a concise discussion on the impact of CFA-induced craniofacial inflammation on anxiety-like responses. We then explore the therapeutic and preventive effects of agmatine on these behaviors, along with the associated epigenetic changes and neural activities.

### 4.1. Effects of Craniofacial Inflammation on Anxiety-like Responses

In the vehicle-treated CFA group, multiple behavioral tests consistently revealed increased anxiety-like behaviors over time compared to pre-CFA (Pre) levels. These results align with prior studies employing craniofacial inflammation models [22,48,49], as well as models involving inflammation outside the trigeminal system, including systemic [50] and pulmonary inflammation [51]. Furthermore, the NOR results suggest that persistent craniofacial inflammation caused cognitive impairments, consistent with previous reports [29,30,52].

To further characterize the neural basis of these behavioral changes under persistent craniofacial inflammation, we examined epigenetic changes and neural activity in multiple brain regions known to regulate anxiety-like responses. Immunohistochemical analysis revealed increased expression of acetylated histone H3, FosB, and c-Fos in the ACC, IC, RVM, and C2. These changes are consistent with prior reports demonstrating an inflammation-induced upregulation of FosB and histone acetylation in the IC [53], and increased c-Fos in the RVM [54] and C2 [23].

Importantly, our data extend these findings by showing simultaneous, widespread activation of molecular markers across multiple brain regions 12 days after CFA injection, providing a more integrative view of how persistent craniofacial inflammation impacts central circuits involved in emotional regulation. This widespread neural plasticity supports and expands on previous work showing that craniofacial pain-like behaviors in this model extend beyond the craniofacial region, affecting distant sites such as the hindpaw [23].

Taken together, these findings confirm the robustness of the craniofacial inflammation model as a platform for investigating inflammation-induced anxiety, both behaviorally and at the molecular level. The broad distribution of epigenetic and activity-related markers in the brain serves as a valuable baseline for assessing the therapeutic potential of compounds such as agmatine in reversing inflammation-induced neural changes.

These data provide a comprehensive reference of inflammation-driven behavioral and neural changes and set the stage for evaluating targeted interventions aimed at normalizing these maladaptive processes.

### 4.2. Therapeutic Effects of Agmatine on Anxiety-like Behaviors

The therapeutic administration of agmatine (AGMt) attenuated anxiety-like behaviors in mice with persistent craniofacial inflammation. Across multiple behavioral paradigms, including the EPM, DL, OF, and SI tests, anxiety-like behaviors in AGMt-treated CFA mice were similar to the Pre level at each time point. However, they showed a decrease when compared to vehicle-treated mice at each time point after CFA injection. Notably, these anxiolytic effects were observed from Days 3–11 post-injection, with the 30 mg/kg dose eliciting consistent behavioral normalization across tests, while the 1.0 mg/kg dose produced slightly delayed but still statistically significant effects. For example, in the SI test, 30 mg/kg agmatine-treated mice maintained social interaction levels comparable to the Pre level throughout the testing period (Figure 4D), while the EPM and OF tests showed a recovery of exploratory behavior, indicated by increases in the time spent in the open arms and center areas, respectively, at multiple time points (Figure 2B and Figure 3B). These results suggest a broad and sustained anxiolytic profile of mice with persistent craniofacial inflammation.

Locomotor activity, as measured by the total movement distance in the OF test (Figure 3C, upper panel), was restored in CFA mice treated with 1.0 and 30 mg/kg AGMt on Days 8 and 11 compared to the Pre level. Notably, agmatine did not alter locomotor activity in non-CFA mice in the present study. These findings suggest that agmatine selectively ameliorates inflammation-induced behavioral abnormalities without affecting general motor function.

**Figure 10:**
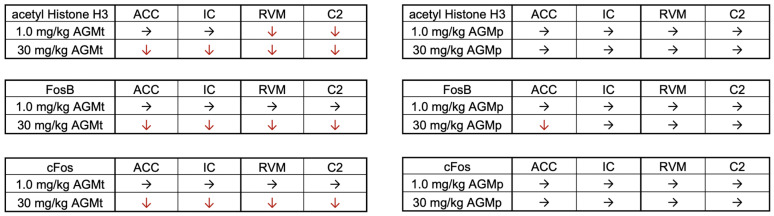
Summary of results showing the effects of therapeutic (AGMt) and preventive (AGMp) administration on acetylated histone H3, FosB, and c-Fos responses in various brain regions relative to the vehicle-treated group on Day 12 after CFA injection. The downward arrow (↓) indicates a significant decrease in the number of positive cells compared with the vehicle-treated CFA group. The rightward arrow (→) indicates no significant change compared with the vehicle-treated CFA group. Abbreviations. ACC, anterior cingulate cortex; IC; insular cortex, RVM, rostral ventromedial medulla; C2, upper cervical spinal dorsal horn.

Corresponding to behavioral recovery, IHC analysis revealed a normalization of neural activity and epigenetic markers in key anxiety-related brain regions in CFA mice. Therapeutic agmatine administration significantly reduced the expression of acetylated histone H3, FosB, and c-Fos in the ACC, IC, RVM, and C2, particularly at the dose of 30 mg/kg (Figure 10, left). These regions are critically involved in processing emotion-related responses under inflammatory conditions [55,56,57].

The effect of 1.0 mg/kg agmatine was less compared to 30 mg/kg agmatine, with observable molecular changes restricted to the RVM and C2, suggesting a dose- and region-dependent action. This pattern mirrors previous findings showing that agmatine exerts site-specific regulatory effects depending on the severity and duration of inflammation [4,7].

These behavioral and molecular findings together point to a mechanistically supported anxiolytic effect of agmatine, likely involving reducing neural responses in the brain through epigenetic regulation and immediate-early gene modulation. Our current results have not revealed the precise mechanism; however, several explanations might be available, and mechanistically, the anxiolytic effects of agmatine are likely multifaceted.

A primary mechanism involves the antagonism of NMDA receptors, particularly GluN2B subunits [58,59]. Craniofacial inflammation is known to enhance excitatory glutamatergic signaling in regions such as the ACC and IC, and agmatine’s blockade of NMDA receptor activity might effectively dampen this pathological hyperexcitability.

In addition to its direct receptor-mediated effects, agmatine might exert prolonged anxiolytic actions through its metabolic conversion into polyamines. Upon decarboxylation, agmatine generates putrescine [60], a polyamine that possesses antioxidative and anti-inflammatory properties [61]. Furthermore, putrescine can be metabolized into 4-aminobutanal and subsequently into GABA, a key inhibitory neurotransmitter known to suppress anxiety-like and depressive behaviors [62]. Although the in vivo half-life of putrescine in the brain remains unclear, this pathway might also contribute to the sustained enhancement of an inhibitory tone following agmatine treatment.

These mechanisms suggest that agmatine not only directly reduces pathological excitatory signaling via NMDA receptor antagonism but also indirectly promotes inhibitory signaling through polyamine and GABA metabolism. This dual-action model provides a coherent explanation for the broad and lasting anxiolytic effects observed in persistent craniofacial inflammation, at least in part.

It is well documented that decreases in glutamatergic tones and increases in GABA tones have been associated with decreases in the neural activities of FosB and c-Fos expressions [63,64]. Furthermore, in the case of acetylated histone H3, while glutamatergic stimulation (e.g., via NMDA receptors) can induce an increase in its acetylation [65], the observed reduction in acetylated histone H3 expression in the present study is consistent with agmatine’s known modulatory effects on glutamatergic and GABAergic systems [66,67]. These linkages need to be evaluated in future studies.

The ability of the therapeutic administration of agmatine to normalize histone H3 acetylation aligns with growing evidence that chronic inflammation induces epigenetic dysregulation in anxiety-related brain circuits, contributing to persistent anxiety-like responses [31,32].

The observed reductions in histone H3 acetylation, as well as FosB and c-Fos expressions, by the therapeutic administration of agmatine might reflect a reversal of neural changes associated with inflammation. These molecular changes, coupled with behavioral improvements, support the notion that agmatine might reverse inflammation-induced neural changes in the brain.

Further, 30 mg/kg AGMt treatment normalized cognitive behaviors in CFA mice as measured by the NOR test on Days 8 and 11 (Figure 5). The weaker effects observed on Days 2 and 4 might be attributable to the acute and severe inflammation in the craniofacial tissues, which agmatine might not be sufficient to counteract at these early time points. A preclinical report demonstrated that systemic administration of 40 mg/kg agmatine improved spatial reference learning and memory functions in aged rats [68].

Collectively, these findings highlight the selective effect of agmatine, a desirable property for potential therapeutic applications in anxiety-like responses under conditions of persistent craniofacial inflammation.

### 4.3. Preventive Effects of Agmatine on Anxiety-like Behaviors

While our data support a potential role for preventive agmatine administration (AGMp) in attenuating anxiety-like behaviors under persistent craniofacial inflammatory conditions, the effects were generally modest, time-dependent, and varied across behavioral paradigms, compared to the results of AGMt effects.

In the DL test, a transient anxiolytic effect of 30 mg/kg AGMp was observed only on Day 1, with no change in the time spent in the light compartment on Days 3, 7, and 10 (Figure 2D, lower panel). In contrast, the EPM test revealed anxiolytic effects on Days 7 and 10, but not on Days 1 or 3 (Figure 2B, lower panel). Similarly, in the OF test, the time spent in the center area was comparable to the Pre level on Days 4, 8, and 11, but not on Day 2 (Figure 3B, lower panel). These patterns imply that AGMp’s preventive effects are delayed and behavioral-test-specific, manifesting during the persistent phase of inflammation.

In the SI test, although full behavioral recovery was not achieved at either dose, partial improvements were evident in CFA mice: notably, treatment with 30 mg/kg AGMp considerably extended the time spent in the interaction zone on Days 1 and 3 compared to vehicle treatment. These results indicate that the social aspects of anxiety were only partially ameliorated by preventive agmatine administration. While AGMp promoted modest improvements in social interaction behaviors, it failed to fully restore behavior to the Pre levels. This finding suggests that the neural circuits underlying social behaviors might be less responsive to preventive agmatine effects than those mediating general exploratory or avoidance behaviors, a notion consistent with previous findings [69,70].

Moreover, results from the NOR test indicate that AGMp failed to prevent CFA-induced impairments in recognition memory. Mice treated with AGMp continued to spend a shorter time discovering the new object during Test 2 (Figure 5B), suggesting a lack of sustained cognitive benefit following AGM treatment cessation. This dissociation between cognitive behaviors assessed by the NOR test and anxiety-like behaviors, assessed through the EPM, SI, OF, and DL tests, might reflect the differential engagement of neural substrates, as previously reported in chronic psychological stress models [69].

Locomotor activity remained stable in AGMp-treated CFA mice, with the total movement distance across all testing days maintained at the Pre level. Interestingly, mice in the AGMt (therapeutic) group exhibited a delayed recovery of locomotion on Days 8 and 11, with reduced movement noted on Days 2 and 4. These findings suggest that AGMp might have preserved normal locomotor activity, potentially through the modulation of inflammatory responses [71,72]. However, the precise mechanisms remain to be clarified.

Compared to the behavioral results, the effects of AGMp on brain molecular markers were relatively limited. AGMp induced only minor changes in acetylated histone H3 and c-Fos expression across the brain regions examined 12 days after CFA administration. A notable exception was the selective reduction in FosB-positive cells in the ACC on Day 12. This region- and marker-specific response is consistent with previous reports suggesting that preventive agmatine treatment may lead to subtle neurobiological adaptations related to FosB expression, without inducing prominent epigenetic changes [6,73].

These findings suggest that agmatine might exert anxiolytic effects by modulating neural excitability or plasticity in specific regions such as the ACC. Overall, the region-specific molecular profiles and the discrepancy between behavioral and molecular outcomes, particularly evident on Day 12, indicate that AGMp produces only partial anxiolytic effects. These effects are likely mediated by alternative or delayed mechanisms not fully captured by the molecular markers assessed in this study. This represents a limitation, as the study focused only on one post-treatment time point, leaving it unclear whether more prominent molecular changes might occur at earlier or later stages.

In addition to the mechanisms discussed above, potential explanations for the observed delayed anxiolytic effects include non-epigenetic pathways, such as a modulation of nitric oxide signaling, monoaminergic neurotransmission, or glial cell activity—all processes known to be influenced by agmatine and other dietary bioactives [37,74,75]. However, the exact contribution of these mechanisms to the delayed anxiolytic effects of preventive agmatine treatment under persistent craniofacial inflammation remains to be elucidated.

Finally, the agmatine doses employed in our study were selected based on a substantial body of literature demonstrating anxiolytic effects in animal models, particularly within the range of 1 to 30 mg/kg in mice. Extrapolating these doses to humans (assuming a 70 kg adult) corresponds to approximately 70 to 2100 mg of agmatine. These effective doses are comparable to those reported to exert anti-nociceptive effects in patients with neuropathic pain (2600 mg agmatine) [76].

### 4.4. Limitation

First, our study included only male subjects. This sex bias might limit the generalizability of our findings, as drug responses and local pathological conditions are well known to differ between males and females. Second, we assessed neural function during the chronic phase (Day 12) of inflammation but did not examine the acute phase (Days 1–4). Although chronic inflammation is clinically relevant, investigating the acute stage might yield additional insights into the role of craniofacial inflammation in anxiety-like behaviors. Third, while we evaluated anxiety-like behaviors in relation to epigenetic changes and neural activity, the specific molecular mechanisms underlying these changes remain largely unexplored.

### 4.5. Future Perspective: Dietary Agmatine from Sake Lees Extracts

Our metabolomic analysis revealed that sake lees, previously shown to reduce depression-like behaviors in rats [77], contained approximately 0.37 mM agmatine. Given that 0.8 mM agmatine (0.03 mg agmatine (FW = 130) in 0.3 mL/mouse) corresponds to an effective dose of 1.0 mg/kg agmatine administered in our experiments, we estimate that approximately 0.5 mL of sake lees would be required to deliver this dose per mouse under the same conditions. Although we did not evaluate the functional effects of sake lees directly, the confirmed presence of agmatine raises the possibility that the consumption of agmatine-rich fermented foods may modulate inflammation-related emotional states. Future studies are warranted to examine the bioavailability and efficacy of food-derived agmatine in modulating anxiety-like behaviors under craniofacial inflammatory conditions.

## 5. Conclusions

Therapeutic agmatine administration reduced anxiety-like behaviors and normalized three key markers in anxiety-related brain regions under persistent craniofacial inflammation. Furthermore, preventive treatment also showed mild but significant effects on anxiety-like behavioral and neural responses. This is the first report on the beneficial effects of agmatine, contained in various foods, including sake, on anxiety-like responses evoked by conditions of persistent craniofacial inflammation.

## Data Availability

The original contributions presented in the study are included in the article/Supplementary Materials, further inquiries can be directed to the corresponding author.

## Supplementary Materials

The following supporting information can be downloaded at https://www.mdpi.com/article/10.3390/nu17111848/s1, Figure S1: Effects of persistent craniofacial inflammation on craniofacial pain-like behaviors in mice; Figure S2: Effects of agmatine administration on novel object recognition behaviors; Table S1: Sample size utilized in each behavioral assessment; Table S2: Sample size utilized in the immunohistochemical experiments.
