# Supplementary material for: Daily Administration of Agmatine Reduced Anxiety-like Behaviors and Neural Responses in the Brains of Male Mice with Persistent Inflammation in the Craniofacial Region
Source: Nutrients. 2025 May 28;17(11):1848. doi: 10.3390/nu17111848 (PMC12158226; doi:10.3390/nu17111848)
Supplement: Supplementary file 1 [file nutrients-17-01848-s001.zip › nutrients-3636230-supplementary.pdf]

Supplementary Figure S1

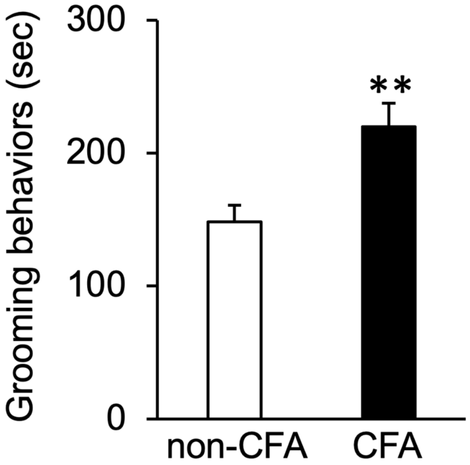

Figure S1. Effects of persistent craniofacial inflammation on craniofacial pain-like behaviors in male C57BL/6J mice. This study examined the effect of persistent craniofacial inflammation, induced by CFA injection into the left masseter muscle, on craniofacial pain-like behaviors following a 2.5% formalin (5 μL) injection into the skin over the same muscle. Grooming behavior directed at the affected area was recorded for 30 min on Day 12 after CFA injection. Results were compared between non-CFA groups (n = 10) and CFA-treated (CFA group, n = 10) ($F(1, 18) = 108.7$, $p < 0.0001$). Notably, the findings are consistent with previous reports, demonstrating that this CFA model exhibited elevated craniofacial pain-like behaviors. ** $p < 0.0001$ vs. non-CFA group.

Supplementary Figure S2

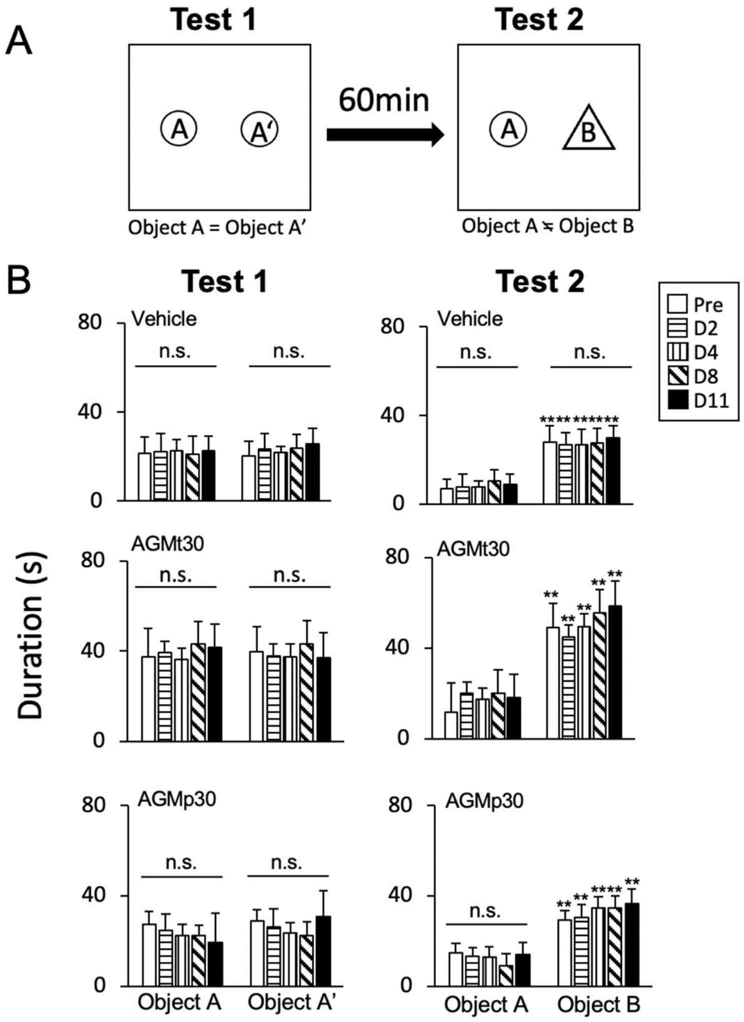

Figure S2. Experimental procedures (**A**) and summary data (**B**) for the effect of agmatine (AGM) on the ability of novel object recognition (NOR) in the non-CFA group. There was no significant main effect of the time course (Pre, Days 2, 4, 8, 11) in vehicle ($F(4, 144) = 0.7$, $p < 0.58$), 30 mg/kg AGMt ($F(4, 152) = 2.18$, $p < 0.74$). The 30 mg/ kg AGMp exhibited significant main effects of the time course ($F(4, 144) = 3.09$, $p < 0.02$); however, post hoc tests revealed no significant differences across all comparisons ($p < 0.1$). There was significant main effect of the region (object) in vehicle ($F(3, 36) = 8.54$, $p < 0.0001$) and 30 mg/ kg AGMt ($F(3, 36) = 23.79$, $p < 0.0001$), and 30 mg/kg AGMp ($F(3, 26) = 84.1$, $p < 0.0003$). ** $p < 0.0001$ versus pre-treatment (Pre) within each group; b, $p < 0.0001$ versus the vehicle group on the corresponding day; n.s., no significance.

Supplementary Table S1. Sample size in each behavioral test. Total 118 mice were used for the assessments of anxiety-like behaviors using EPM, DL, OF, SI, and NOR tests. Results obtained from vehicle group shown in the EPM test was employed as controls in the other test. In a separate experiment (*), twenty mice were employed to assess orofacial pain-like behaviors (orofacial formalin test).

EPM Test	Non-CFA	CFA
Vehicle	10	11
AGMt 1.0mg/kg	8	10
AGMt 30mg/kg	10	10
AGMp 1.0mg/kg	9	10
AGMp 30mg/kg	10	10
Total	47	51

DL test	Non-CFA	CFA
AGMt 1.0mg/kg	8	10
AGMt 30mg/kg	10	10
AGMp 1.0mg/kg	9	10
AGMp 30mg/kg	10	10
Total	37	40

OF test	Non-CFA	CFA
AGMt 1.0mg/kg	8	10
AGMt 30mg/kg	10	10
AGMp 1.0mg/kg	9	10
AGMp 30mg/kg	10	10
Total	37	40

SI test	Non-CFA	CFA
AGMt 1.0mg/kg	8	10
AGMt 30mg/kg	10	10
AGMp 1.0mg/kg	9	10
AGMp 30mg/kg	10	10
Total	37	40

NOR test	Non-CFA	CFA
AGMt 30mg/kg	10	10
AGMt 30mg/kg	10	10

Formalin test *	Non-CFA	CFA
No drug	10	10

Supplementary Table S2. Sample Size for immunohistochemical (IHC) experiments. A total of 76 mice were used to assess immunoreactivity for acetylated histone H3, FosB, and c-Fos in various brain regions. These mice underwent IHC following the completion of behavioral assessments. Data from the vehicle-treated group (*) were used as controls for comparisons in other tests.

ACC		acetyl Histone H3
	Non-CFA	CFA
Vehicle*	9	8
AGMt 1.0mg/kg	7	5
AGMt 30mg/kg	8	8
AGMp 1.0mg/kg	6	6
AGMp 30mg/kg	9	7
Total	39	34

IC		acetyl Histone H3
	Non-CFA	CFA
Vehicle *	9	8
AGMt 1.0mg/kg	7	5
AGMt 30mg/kg	8	8
AGMp 1.0mg/kg	6	6
AGMp 30mg/kg	9	7
Total	39	34

ACC		FosB
	Non-CFA	CFA
AGMt 1.0mg/kg	7	6
AGMt 30mg/kg	8	8
AGMp 1.0mg/kg	7	7
AGMp 30mg/kg	9	7
Total	31	28

IC		FosB
FosB	Non-CFA	CFA
AGMt 1.0mg/kg	7	6
AGMt 30mg/kg	8	8
AGMp 1.0mg/kg	7	7
AGMp 30mg/kg	9	7
Total	31	28

ACC		c-Fos
FosB	Non-CFA	CFA
AGMt 1.0mg/kg	7	7
AGMt 30mg/kg	8	8
AGMp 1.0mg/kg	7	6
AGMp 30mg/kg	9	7
Total	31	28

IC		c-Fos
FosB	Non-CFA	CFA
AGMt 1.0mg/kg	7	7
AGMt 30mg/kg	8	8
AGMp 1.0mg/kg	7	6
AGMp 30mg/kg	9	7
Total	31	28

RVM		acetyl Histone H3
	Non-CFA	CFA
Vehicle *	9	8
AGMt 1.0mg/kg	7	7
AGMt 30mg/kg	8	8
AGMp 1.0mg/kg	7	6
AGMp 30mg/kg	9	7
Total	40	36

C2		acetyl Histone H3
	Non-CFA	CFA
Vehicle *	9	8
AGMt 1.0mg/kg	7	7
AGMt 30mg/kg	8	8
AGMp 1.0mg/kg	7	6
AGMp 30mg/kg	9	7
Total	40	36

RVM		FosB
	Non-CFA	CFA
AGMt 1.0mg/kg	7	7
AGMt 30mg/kg	8	8
AGMp 1.0mg/kg	7	6
AGMp 30mg/kg	9	7
Total	31	28

C2		FosB
FosB	Non-CFA	CFA
AGMt 1.0mg/kg	7	7
AGMt 30mg/kg	8	8
AGMp 1.0mg/kg	7	6
AGMp 30mg/kg	9	7
Total	31	28

RVM		c-Fos
	Non-CFA	CFA
AGMt 1.0mg/kg	7	7
AGMt 30mg/kg	8	8
AGMp 1.0mg/kg	7	6
AGMp 30mg/kg	9	7
Total	31	28

C2		c-Fos
	Non-CFA	CFA
AGMt 1.0mg/kg	7	7
AGMt 30mg/kg	8	8
AGMp 1.0mg/kg	7	6
AGMp 30mg/kg	9	7
Total	31	28
